# A Randomized Controlled Trial Evaluating the Effect of Local Application of Sodium Alendronate Gel and Low-Level Laser Therapy (LLLT) On Peri-Implant Tissue Healing in Wistar Rats

**DOI:** 10.12688/f1000research.160479.1

**Published:** 2025-01-30

**Authors:** Divya Mishra, Arun S Urala, Ashwath S Nayak, Divya S, Rajay Kamath, Lakshmi Narayan Bairy, Gargi S

**Affiliations:** 1 Department of Orthodontics and Dentofacial Orthopaedics, Manipal College of Dental Sciences, Manipal Academy of Higher Education, Manipal, Karnataka, 576104, India; 2 Department of Orthodontics and Dentofacial Orthopaedics, Bapuji Dental College and Hospital, Davanagere, Karanataka, 577004, India; 3 Department of Clinical Genomics, Mayo Clinic Minnesota, Rochester, Minnesota, USA; 4 Department of Pharmacology, RAK College of Medical Sciences, RAK Medical and Health Sciences University, Ra’s Al Khaimah, United Arab Emirates

**Keywords:** Micro-implants, anchorage, animal study, Low level laser therapy, photo-biomodulation, bisphosphonates

## Abstract

**Objective:**

To evaluate the effect of local application of Sodium Alendronate incorporated in Carbapol gel together with LLLT on peri-implant tissue healing in Wistar rat femurs.

**Methods:**

Twenty-four male Wistar rats were randomly divided into 4 groups: Group 1 (the control group), Group 2 (the Sodium Alendronate group), Group 3 (the Sodium Alendronate and LLLT group), and Group 4 (the LLLT group). Mini implants were placed in right and left femur bones in all the four groups. Implants of groups 2 and 3 were coated in 1mg Carbapol gel incorporated with 1 mg Sodium alendronate. Groups 3 and 4 were exposed to LLLT (CO _2_ laser, wavelength 830nm; 2.1J/cm ^2^) on 1 ^st,^ 7 ^th^, 14 ^th^ and 21 ^st^ day. Animals were sacrificed on the 28th day, following which the femurs were dissected out and stored in 10% buffered formaldehyde for histopathological analysis.

**Results:**

Groups 3 and 4 showed bony union and formation of reorganized spongiosa whereas Groups 1 and 2 showed fibrous union. The bone marrow from Group 3 had an adult-type fatty marrow, while that from Group 2 and 4 was occupied by red blood cells. Group 1 showed initial stages of bone healing in which the defect occupied more than half of the bone marrow.

**Conclusion:**

LLLT given using CO _2_ laser therapy, together with a one-time application of Sodium Alendronate in Carbapol gel at the time of implant placement optimally enhances healing of peri-implant tissues.

**List of abbreviations**
LLLT: Low Level Laser Therapy
CO _2_
: Carbon di oxide
i.p: Intra peritoneal
®: Registered trademark
mm: Millimeter
mg: Milligram
J/cm ^2^
: Joule per square centimeter
IR: Infra-red
W: Watt
%: Percentage
EDTA: Ethylenediaminetetraacetic acid
H&E: Hematoxylin and Eosin
SPSS: Statistical Package for the Social Sciences
ANOVA: Analysis of Variance
nm: Nanometer
GaAlAs: Gallium-aluminum-arsenide
mW: Milli watt
TGFβ-1: transforming growth factor beta-1
sec: Seconds
SPSS: Statistical Package for Social Sciences
<: Lesser than
>: Greater than

## Clinical significance

Mini-implant used as stationary anchorage tends to fail due to peri-implantitis or poor bone quality and many other reasons. As these mini-implants derive support from cortical bone and not via osseointegration, achieving their stability for a long run has become a prime concern. In this study, we have come up with an alternate way which can be followed for improving the stability of the implants. The practice of using mini-implants is now getting into the mainstream and hence we have done many studies pertaining to the same. The novel method was evaluated by conducting study on Wistar rats.

## Background

Skeletal anchorage has gained popularity in orthodontic practice, particularly in situations where 75% or more space is needed for retraction of anterior teeth - termed critical anchorage - especially when a successful treatment outcome is expected or desired. ^1^ Miniscrew placement is one of the easiest ways to achieve skeletal anchorage for anterior tooth retraction, however, stability and patient safety following orthodontic loading of miniscrews determine overall success of orthodontic treatment. ^2^

Following implant placement, healthy tissues around the implant pose as biological barriers to many intraoral microorganisms that have the propensity to cause inflammation. ^3^ Inflammation of peri-implant tissues, known as peri-implantitis, has the potential to increase implant failure by 30%. ^4^ Peri-implantitis features inflammation of bone around the implant, bleeding on probing, epithelial infiltration and, in some cases, suppuration, which inadvertently leads to failure. ^5^ Furthermore, bone around the neck of screw implants can be lost due to inflammation. ^6, 7^ These are serious problems that need to be addressed at the cellular level to facilitate bone repair in order for implant placement to be successful. To that end, adjunctive treatments like Low-Level Laser Therapy (LLLT) and the local application of bisphosphonates have been considerably employed to optimally enhance peri implant healing.

The biostimulatory effect of LLLT can be considered ‘photostimulatory’ or ‘photomodulatory’ when both nucleic acid formation and cell division increase. ^8, 9^ LLLT also has shown to markedly increase production of osteocytes because of its positive effect on the bone matrix. ^10^ It is also observed that damaged bone can be actively repaired with LLLT irradiation. ^11^

On the other hand, Bisphosphonates are pyrophosphate analogs which are stable and can affect bone matrix formation. They can stimulate proliferation and differentiation of osteoblasts, which in turn enhances bone formation, while inhibiting catabolic osteoclast activity. ^12^ These advantages make bisphosphonates the therapy of choice in settings of osteolytic disease and osteoporosis. ^13^ Notably, Sodium Alendronate, which positively affects osteoblast maturation, has become the most commonly used bisphosphonate, ^14, 15^ however, its systemic administration is precluded when healing of bone around the implant is specifically needed, though it has shown to increase bone formation. Side effects to its use include gastrointestinal disorders and nephrocalcinosis. ^16^ Furthermore, previous studies have reported severe pain and tissue necrosis at the site of injection. ^17^ Hence, local drug delivery of Sodium alendronate at the injury site is considered safe. ^18^

Various studies have reported both LLLT and Bisphosphonates have beneficial effect on bone repair, ^19^ but none of the studies have evaluated the combined effects of LLLT and local Bisphosphonate therapy on the healing of peri-implant tissues. Hence, our study aims at filling in the knowledge gap. Here, we evaluate the combined effect of local application of Sodium Alendronate incorporated in Carbapol gel and Low Level Laser Therapy on peri-implant tissue healing in Wistar rats.

## Methods

Twenty-four male Wistar rats, 6-7 months of age, and weighing about 250-300 g each, were used in this study. Approval was obtained from the Institutional Animal Ethics Committee (IAEC/KMC/11/2016) before the commencement of the study.

The sample size (n) is calculated according to the formula ^20^:

$$n=z^{2*}\;p^{*}\;\left(1-p\right)/e^{2}$$

where,

z = 1.96 for a confidence level (α) of 95%,

p = proportion (expressed as a decimal),

e = margin of error.

z=1.96,p=0.015,e=0.05

On substitution with the values

$$n=1.96^{2*}\;0.015^{*}\;\left(1-0.015\right)/0.05^{2}$$

n=0.0568/0.0025=22.704

n≈23

The calculated sample size was 23 rounded to 24; 6 in each group were considered

N=6

Animals were randomly divided into 4 groups ( Flowchart 1).

**Flowchart 1.**
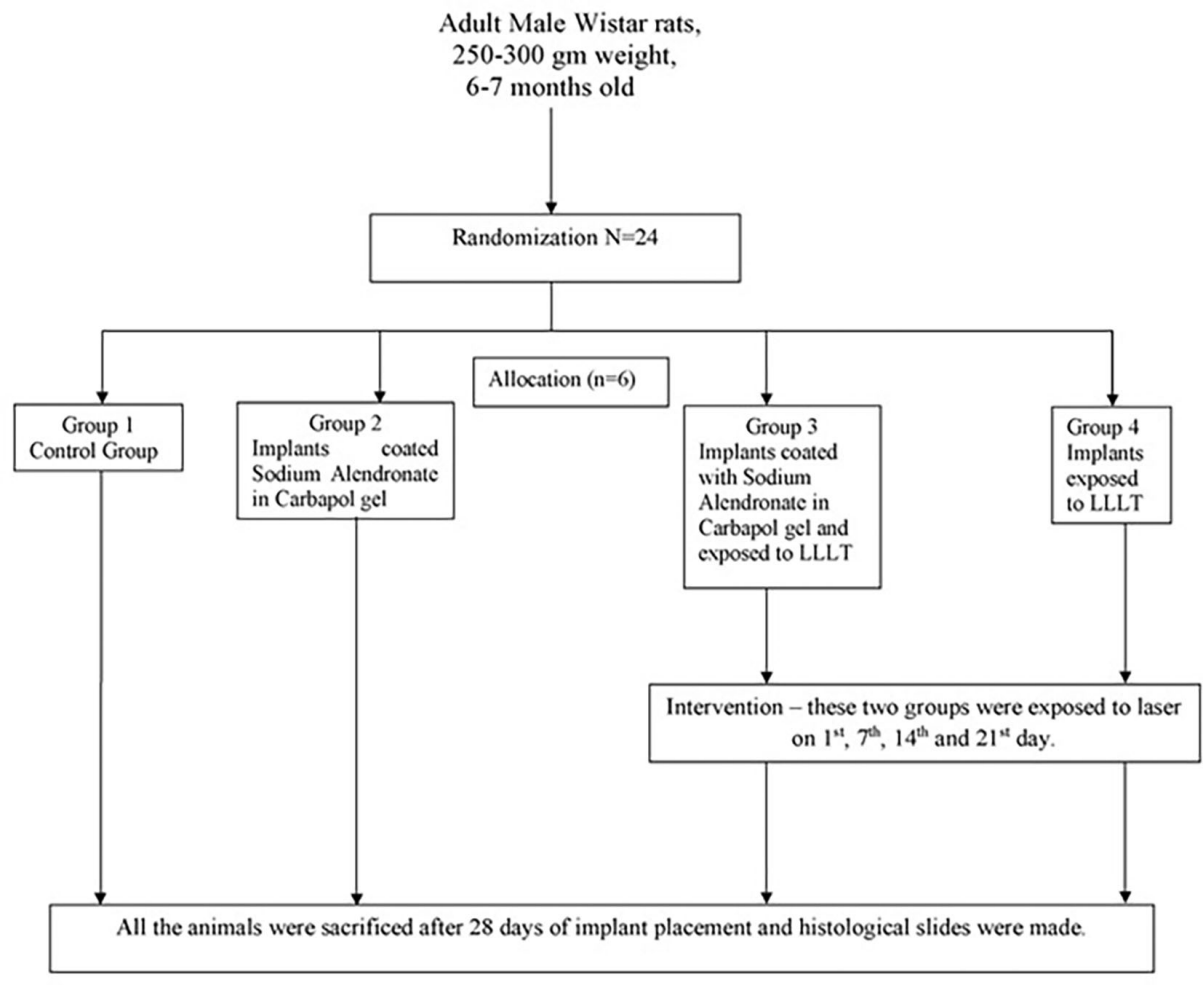
Animals divided randomly into 4 groups.

**Figure 1.**
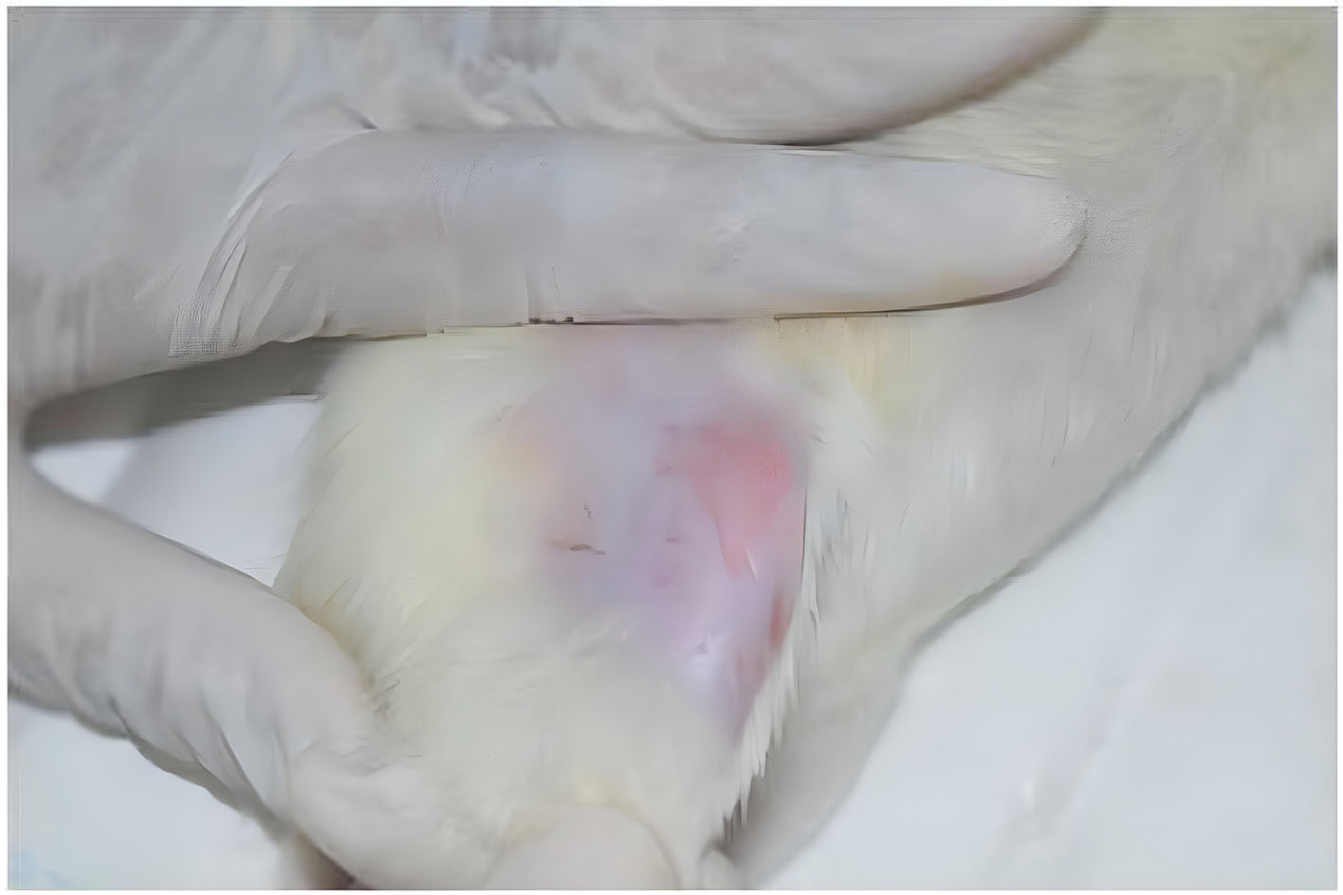
Animals positioned on their lateral decubitus position and lateral part of posterior limb was shaved.

This is a double-blind study. Placement of implants was carried out in all the four groups, after the animals were anaesthetized with thiopentone sodium (45 mg/kg, i.p., Thiosol sodium® Neon Laboratories Limited). ^21– 23^ After the animal was placed in the lateral decubitus position, ^24^ the lateral portion of the posterior limb was shaved ( Figure 1).

**Figure 2.**
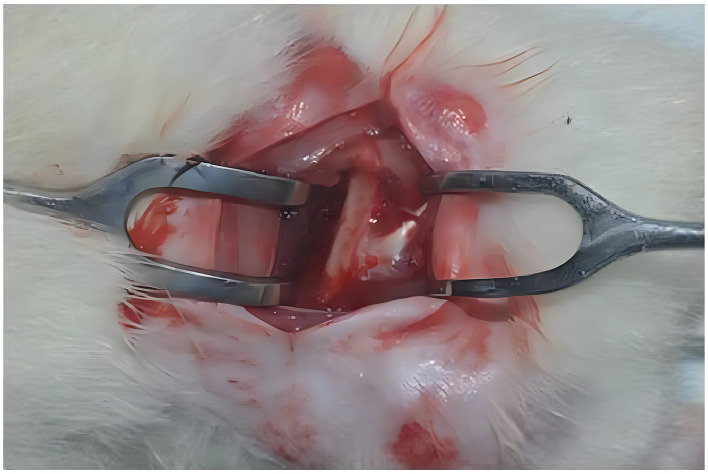
Femur bone exposed and two muscle groups were separated.

**Figure 3.**
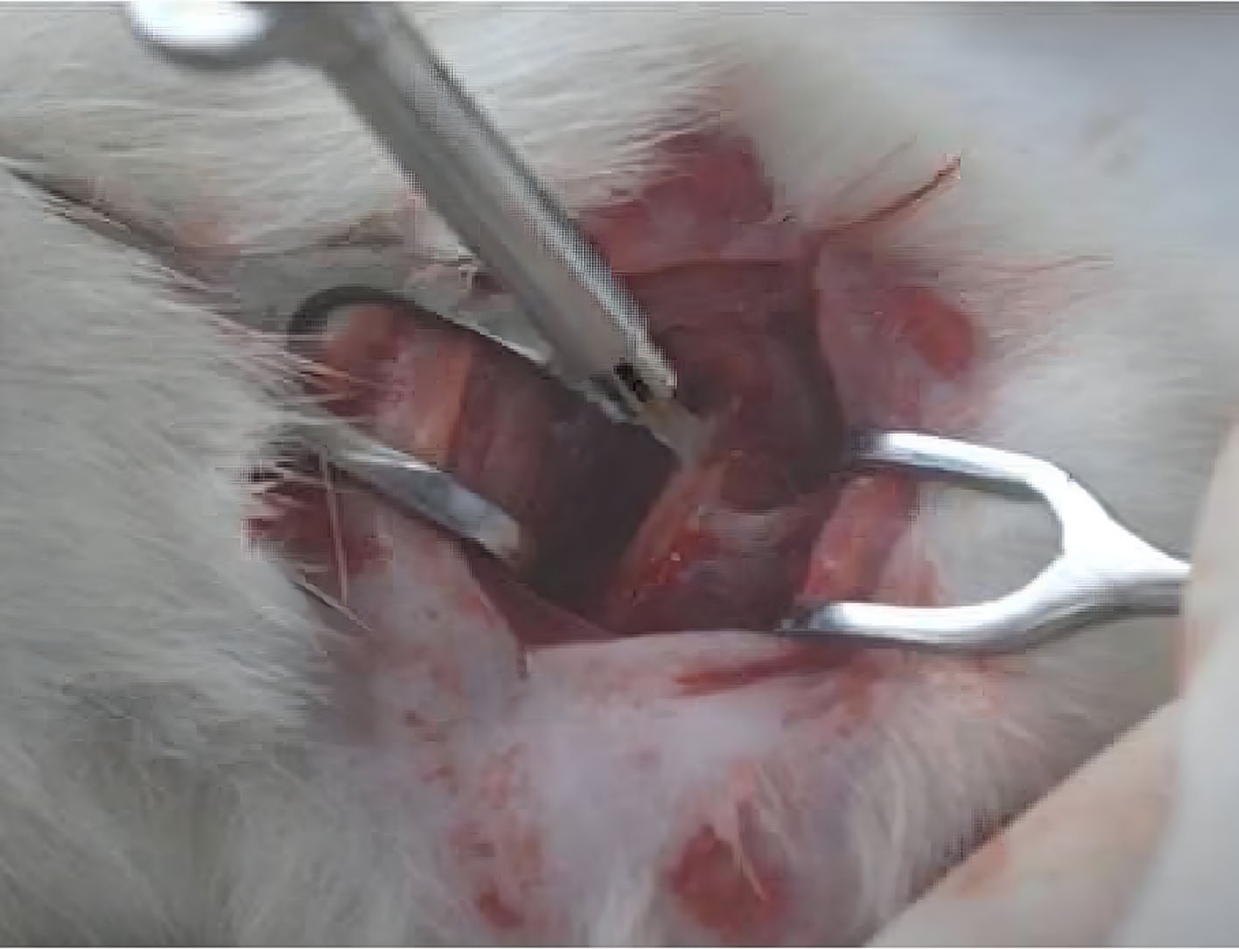
Implant placed on widest portion of femur bone.

Under sterile aseptic conditions, a surgical incision was made, extending from the femoro-tibio-patellar joint (the distal reference point) to the major trochanter (the proximal reference point). Following the exposure of the deeper subcutaneous layers, the intermuscular septum - a thin white line which separates the biceps femoral and superficial gluteus muscles - was identified. ^24^ The intermuscular septum was incised and the two muscle groups were carefully separated using a pair of dissecting scissors, to expose the femur ( Figure 2). The widest portion of the femur was then chosen for implant placement ^24^ ( Figure 3).

**Figure 4.**
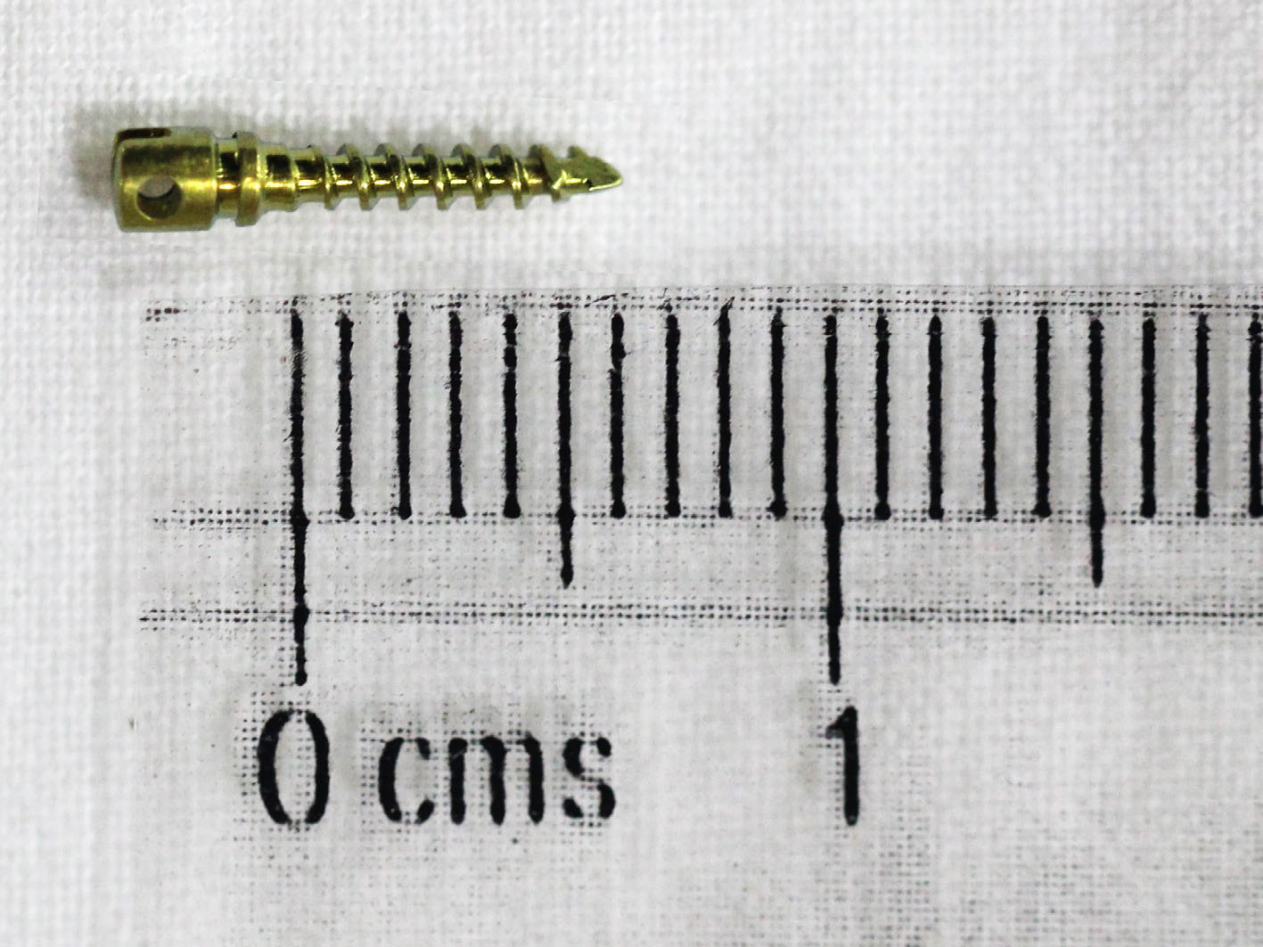
Self-Drilling, titanium miniscrew (M/S S.K. Surgicals) 1.5 × 6 mm.

Self-Drilling, titanium miniscrews (obtained from M/S S.K. Surgicals) of 1.5 × 6 mm in dimension ( Figure 4) were used in this study; one screw was placed on the right femur and one on the left. A manual driver was used to place all the screws monocortically, leaving 2 to 3 threads visible above the surface of the bone. The implants placed in groups 2 and 3 were coated with Carbapol gel containing Sodium Alendronate at a concentration of 1 mg Sodium Alendronate per mg Carbapol gel. Gel phase was preferred to liquid medium, due to increased sustainability in the local area.

**Figure 5.**
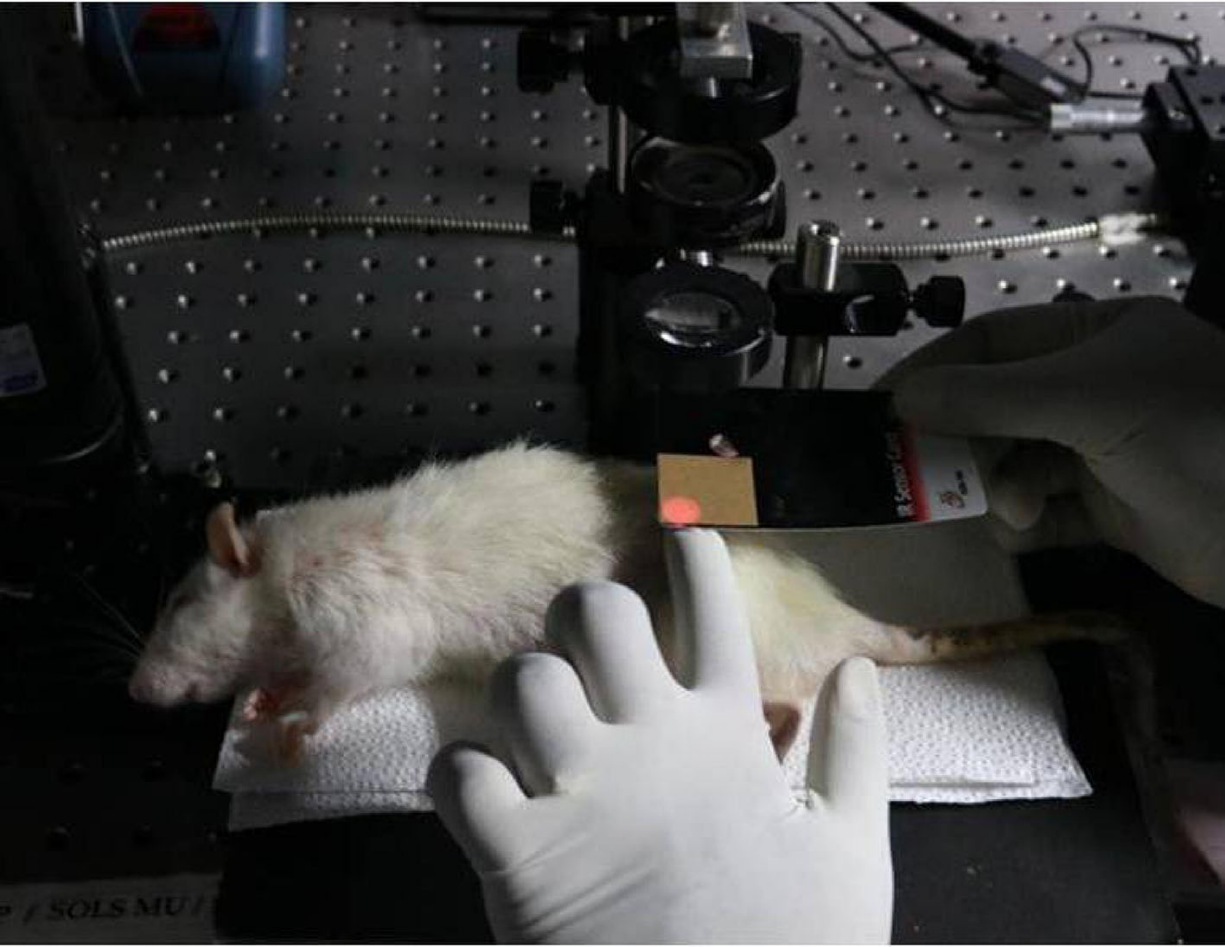
Infra-red card.

Following implant placement, closure was done in layers, with the muscular and skin layers being approximated using catgut (3-O) and silk (3-O) sutures respectively. LLLT was carried out in groups 3 and 4 after implant placement (1st day) and was repeated on the 7 ^th^, 14 ^th^ and 21 ^st^ days with the help of a CO _2_ laser administered at a wavelength of 830 nm. The dose received was about 2.1 J/cm ^2^ (39.2 × 10 ^−3^ W × 55 sec). To obtain maximum exposure of the peri-implant area to the laser beam, the implant head was identified on palpation and marked using an IR (Infrared) card prior to LLLT during the exposure period ( Figure 5). The animals were sacrificed on the 28 ^th^ day with an overdose of anesthesia (Thiopental sodium 50 mg/kg). The right and left femurs were dissected out and fixed in 10% neutral buffered formaldehyde.

For histology, femur decalcification was carried out using 10% EDTA for a period of 6-8 weeks; 5 μ sections were generated after the specimens were embedded in paraffin. Hematoxylin and Eosin staining was done and the sections were examined in a bright field microscope using a 10× objective lens. The amount of healing was evaluated microscopically using a histological scoring system proposed by Heiple et al. ^25^ ( Table 1).

**Table 1.** Histological Scoring System Developed by Heiple et al.

Category	Point
**Union**	Highest score is 4
No sign of union	0
Fibrous union	1
Osteochondral union	2
Bone union	3
Complete Reorganization	4
**Spongiosa**	The highest score is 4
No sign of cellular activity	0
Early bone formation	1
Active new bone formation	2
Reorganized spongiosa formation	3
Complete reorganized spongiosa	4
**Cortex**	The highest score is 4
Absence of cortex	0
Early detection	1
Initiation of formation	2
Reorganization in majority	3
Complete organization	4
**Bone Marrow**	The highest score is 4
Not available	0
Detection of fibrinous material	1
Defect occupying more than half	2
Fully occupying the red bone marrow	3
Adult type fatty marrow	4
**Sum of histologic scores**	16

## Results

Data analysis was done using SPSS software version 20.0 for windows. Intergroup comparison was carried out using one-way ANOVA and further evaluated by Post-hoc Tukey test. Histological results on day 28 revealed that specimens of groups 3 and 4 showed bony union whereas groups 1 and 2 showed fibrous union. There was formation of reorganized spongiosa in groups 3 and 4, while groups 1 and 2 had signs of early bone formation. Cortical reorganization was present in all the groups. Bone marrow examination of group 3 revealed adult type of fatty bone marrow, while that from groups 2 and 4 was occupied by red blood cells. Specimens of group 1 showed initial stages of bone healing in which the defect occupied more than half of the bone marrow cavity.

Statistically, when Post hoc test was applied between the groups on both right and left sides, groups 3 and 4 showed significantly higher scores in terms of union when compared to groups 1 (p = 0.0014, 0.0025) and 2 (p = 0.007, 0.0012) ( Tables 2, 3). Statistically significant differences were found when groups 3 and 4 were compared with group 1 for spongiosa, bone marrow formation and overall histological score ( Tables 2, 3). Compared to the control group, groups 3 and 4 showed reorganized spongiosa with an adult-type fatty bone marrow which is indicative of good healing ( Tables 2, 3). There was a significant difference between groups 3 and 1 (p = 0.015), and group 2 (p = 0.046) in terms of cortical bone organization and formation. No statistically significant differences were seen between groups 3 and 4 with respect to the histological score ( Tables 2, 3). Comparing the scores between the right and left sides, no statistically significant differences were observed ( Table 4).

**Table 2.** statistical analysis between the groups on 28 ^th^ day-left side.

	Group 1	Group 2	Group 3	Group 4	P value (ANOVA test)	Post hoc (between the groups) p value	
Union	2.2±0.447	2.16±0.752	3.76±0.516	3.6±0.547	**0.0001** *	1 vs 2	0.999
						1 vs 3	**0.0014** *
						1 vs 4	**0.0025** *
						2 vs 3	**0.0007** *
						2 vs 4	**0.0012** *
						3 vs 4	0.9614
Spongiosa	2.2±0.447	3±0.0	3.66±0.516	3.4±0.547	**0.0001** *	1 vs 2	0.0191
						1 vs 3	**0.0002** *
						1 vs 4	**0.0006** *
						2 vs 3	0.0987
						2 vs 4	0.3383
						3 vs 4	0.8422
Cortex	3±0.0	3.16±0.408	3.83±0.408	3.6±0.547	**0.0096** *	1 vs 2	0.9020
						1 vs 3	**0.0155** *
						1 vs 4	0.0833
						2 vs 3	**0.0465** *
						2 vs 4	0.2351
						3 vs 4	0.7610
Bone marrow	2.8±0.447	3.62±0.408	4±0.0	3.8±0.447	**0.0002** *	1 vs 2	**0.0060** *
						1 vs 3	**0.0002** *
						1 vs 4	**0.0010** *
						2 vs 3	0.3150
						2 vs 4	0.8132
						3 vs 4	0.7860
Total score	10.2±1.095	12.16±0.752	15.16±1.329	14.4±1.516	**0.0000** *	1 vs 2	0.0645
						1 vs 3	**0.0000** *
						1 vs 4	**0.0001** *
						2 vs 3	**0.0032** *
						2 vs 4	**0.0219** *
						3 vs 4	0.7256

* P<0.05 is significant.

**Table 3.** Statistical analysis between the groups on 28 ^th^ day-right side.

	Control group	Group 2	Group 3	Group 4	P value (ANOVA test)	Post hoc (between the groups) p value	
Union	2.2±0.447	2.33±0.816	3.6±0.816	3.3±0.547	**0.0043** *	1 vs 2	0.9941
						1vs 3	**0.0155** *
						1vs 4	0.0541
						2 vs 3	**0.0192** *
						2 vs 4	0.0697
						3 vs 4	0.8712
Spongiosa	2.4±0.547	2.83±0.408	3.83±0.408	3.4±0.547	**0.0004** *	1 vs 2	0.4202
						1vs 3	**0.0005** *
						1vs 4	**0.0091** *
						2 vs 3	**0.0091** *
						2 vs 4	0.1665
						3 vs 4	0.4202
Cortex	3±0.0	3.23±0.632	4±0.0	3.4±0.547	**0.0076** *	1 vs 2	0.7932
						1vs 3	**0.0060** *
						1vs 4	0.4008
						2 vs 3	**0.0294** *
						2 vs 4	0.8897
						3 vs 4	0.9994
Bone marrow	2.2±0.447	3.66±0.516	4±0.0	3.8±0.447	**0.0000** *	1 vs 2	**0.0000** *
						1vs 3	**0.0000** *
						1vs 4	**0.0000** *
						2 vs 3	0.4078
						2 vs 4	0.9006
						3 vs 4	0.7860
Total score	9.8±0.836	11.83±1.941	15.16±0.983	14.2±1.095	**0.0000** *	1 vs 2	0.0768
						1vs 3	**0.0000** *
						1vs 4	**0.0001** *
						2 vs 3	**0.0023** *
						2 vs 4	**0.0238** *
						3 vs 4	0.6152

* P<0.05 is significant.

**Table 4.** Comparison between scores of left and right side.

		Control group	Group 2	Group 3	Group 4
Union	LEFT	2.2±0.447	2.16±0.752	3.76±0.516	3.6±0.547
	RIGHT	2.2±0.447	2.33±0.816	3.6±0.816	3.3±0.547
	P value (t- test)	1(t=0.0)	0.715(t=0.375)	0.693(t=0.405)	0.364(t=0.949)
Spongiosa	LEFT	2.2±0.447	3±0.0	3.66±0.516	3.4±0.547
	RIGHT	2.4±0.547	2.83±0.408	3.83±0.408	3.43±0.547
	P value (t- test)	0.503(t=0.693)	0.344(t=0.991)	0.540(t=0.633)	0.962(t=0.095)
Cortex	LEFT	3±0.0	3.16±0.408	3.83±0.408	3.6±0.547
	RIGHT	3±0.0	3.23±0.632	4±0.0	3.4±0.547
	P value (t- test)	1(t=0.0)	0.824(t=0.227)	0.335(t=1.020)	0.541(t=0.633)
Bone marrow	LEFT	2.8±0.447	3.62±0.408	4±0.0	3.8±0.447
	RIGHT	2.2±0.547	3.66±0.516	4±0.447	3.8±0.447
	P value (t- test)	0.064(t=2.08)	0.884(t=0.148)	0.914(t=0.08)	1(t=0.0)
Total	LEFT	10.2±1.095	12.16±0.752	15.16±1.329	14.4±1.516
	RIGHT	9.8±0.836	11.83±1.941	15.16±0.983	14.2±1.095
	P value (t- test)	0.493(t=0.711)	0.751(t=0.388)	0.892(t=0.101)	0.811(t=0.175)

## Discussion

Placement of mini-implants is one of the easiest ways to achieve skeletal anchorage in situations of critical anchorage, because patient compliance is not needed, though it has many shortcomings of its own. Peri-implantitis is one of the important factors that contribute to dental implant failure. ^6^ ***Park et al*** demonstrated in their study that bone around mini screws in the neck can be damaged due to inflammation. Prevention of inflammation around screw implants is important for success of the mini implant. ^26^ Therefore, many adjunctive treatments have been applied to facilitate faster healing by reducing inflammation. ***Fujimura et al*** reported in their study that orthodontic movement of tooth as well as root resorption was inhibited by bisphosphonates. Similarly, ***Ortega et al*** concluded that small doses of zolendronate, when applied locally, provided good anchorage and prevented bone loss. Additionally, ***Akoyl et al*** reported that local irrigation with Sodium Alendronate trihydrate (at 1mg/ml) proved to be beneficial for healing. They also reported that Sodium Alendronate and LLLT when employed simultaneously on bone defects in Wistar rats enhanced healing. ^19^

In the present study, 1 mg of Sodium Alendronate was incorporated in 1 mg of Carbapol gel (as the latter increases sustainability of drug) and placed around the thread portion of the implant in groups 2 and 3. It was reported by ***Guimaraes et al*** that the beneficial effect of bisphosphonates is dose-dependent and, when applied at higher concentration to the bone surface, sodium alendronate (at 10mg/g) resulted in unfavourable bone remodeling around the implants. ^27^ Though stimulation of bone healing with the help of LLLT is not exactly understood, it is said to be multifactorial and may include stimulation of angiogenesis, production of collagen, ^28– 30^ proliferation and differentiation of osteogenic cells ^31^ mitochondrial respiration and ATP synthesis. ^32, 33^ In an in vitro study, ***Khadra et al*** reported that LLLT (830nm, 1.5 or 3J/cm ^2^) increases attachment, multiplication, differentiation of cells, and formation of TGFβ-1; thus LLLT can regulate cellular activity of human osteoblast-like cells cultured on titanium implants. ^34^ ***Pretel et al*** showed that the application of a GaAlAs (Gallium, Aluminum, and Arsenide) diode laser directly on bone defects in rats leads to modulation of initial inflammatory response and enhanced healing. ^35^ ***Korany et al*** in their study reported that LLLT increases new bone maturation, on seventh and tenth day after irradiation. ^36^ On the other hand, ***Khadra et al*** in their study reported that LLLT (GaAlAs diode laser, 830nm, 75mW) showed noticeable increase of calcium, phosphorous and proteins at 14 ^th^ as well as 28 ^th^ day, which may increase bone formation in calvaria of rats. ^37^

Therefore, in our study by adhering to same wavelength and almost equal power density (830 nm, 39.2 mw) CO _2_ laser was employed at 7-day intervals (for 55 seconds each) at day 1 following implant placement, and was repeated at the 7 ^th^, 14 ^th^ and 21 ^st^ day. All animals were sacrificed at the end of 28 ^th^ day from implant placement. ***Pinheiro et al*** evaluated effects of laser (830 nm, 4 J/cm ^2^) on defects of bone grafted with inorganic bovine bone on femur of Wistar rats; it was reported that LLLT was beneficial for the repair of bone defects when a graft was placed. ^38^ They reported that on the 30 ^th^ day there was presence of high collagen fiber formation which signified mature bone formation. ^38^ Many studies suggested that a dose of 1-5 J/cm ^2^ of LLLT is successful in producing beneficial effects in soft tissues as well as in bone. ^38– 44^ A lower wavelength is prone to dispersion and does not penetrate deep into skin compared to a higher wavelength. ^45^ In one study, it was reported that a laser wavelength of 632 nm penetrates 0.5-1 mm before 37% intensity was lost. ^46^ ***Pinherio et al*** reported that only some percentile of energy is lost before infra-red wavelength penetrates 2 mm deep into the skin. ^34^ Therefore, infra-red (IR) laser light at a wavelength of 630 nm was used in the present study for better bone penetration.

**Figure 6.**
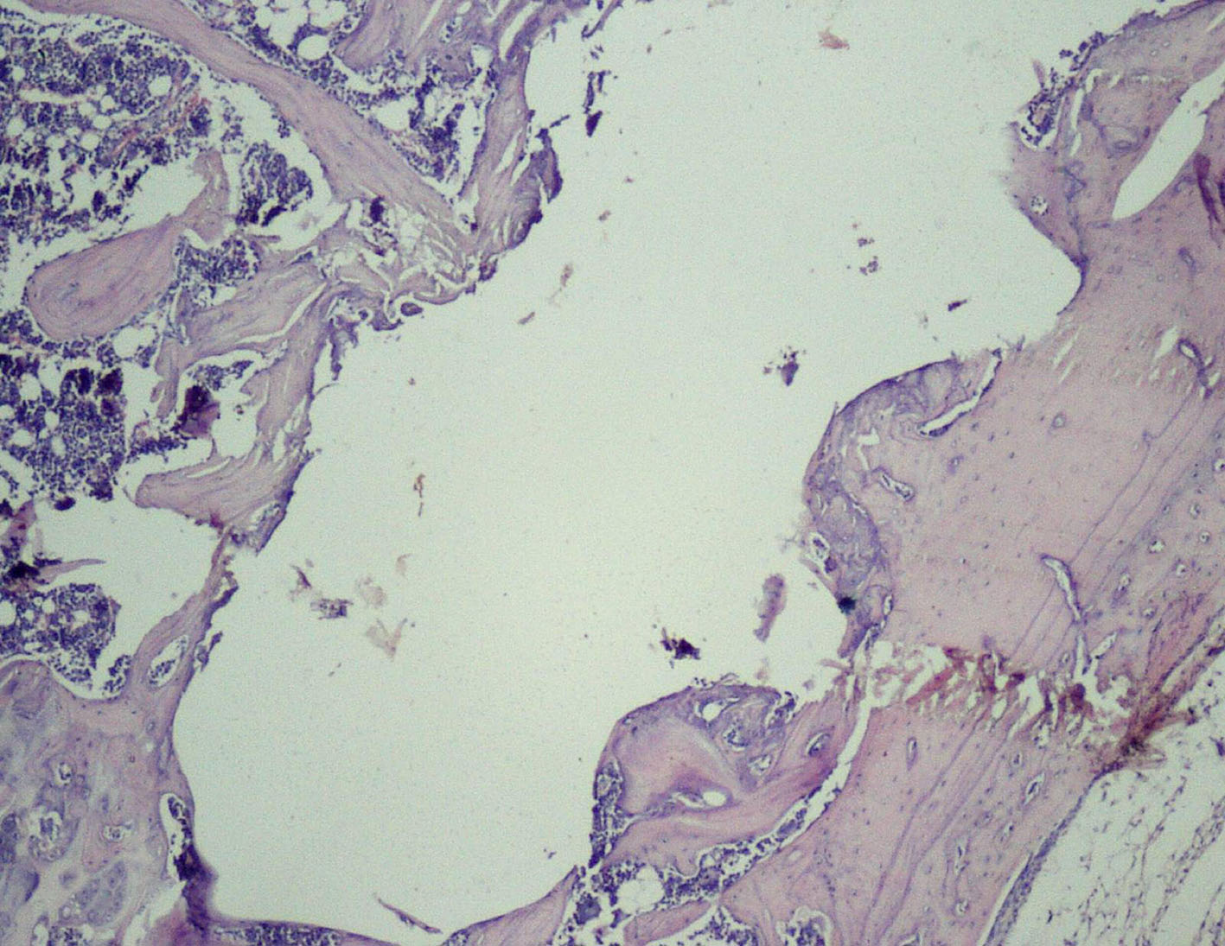
Day 28 of group 3 implant surface with bone formation (photomicrograph of H&E stained slides, original magnification ×10).

**Figure 7.**
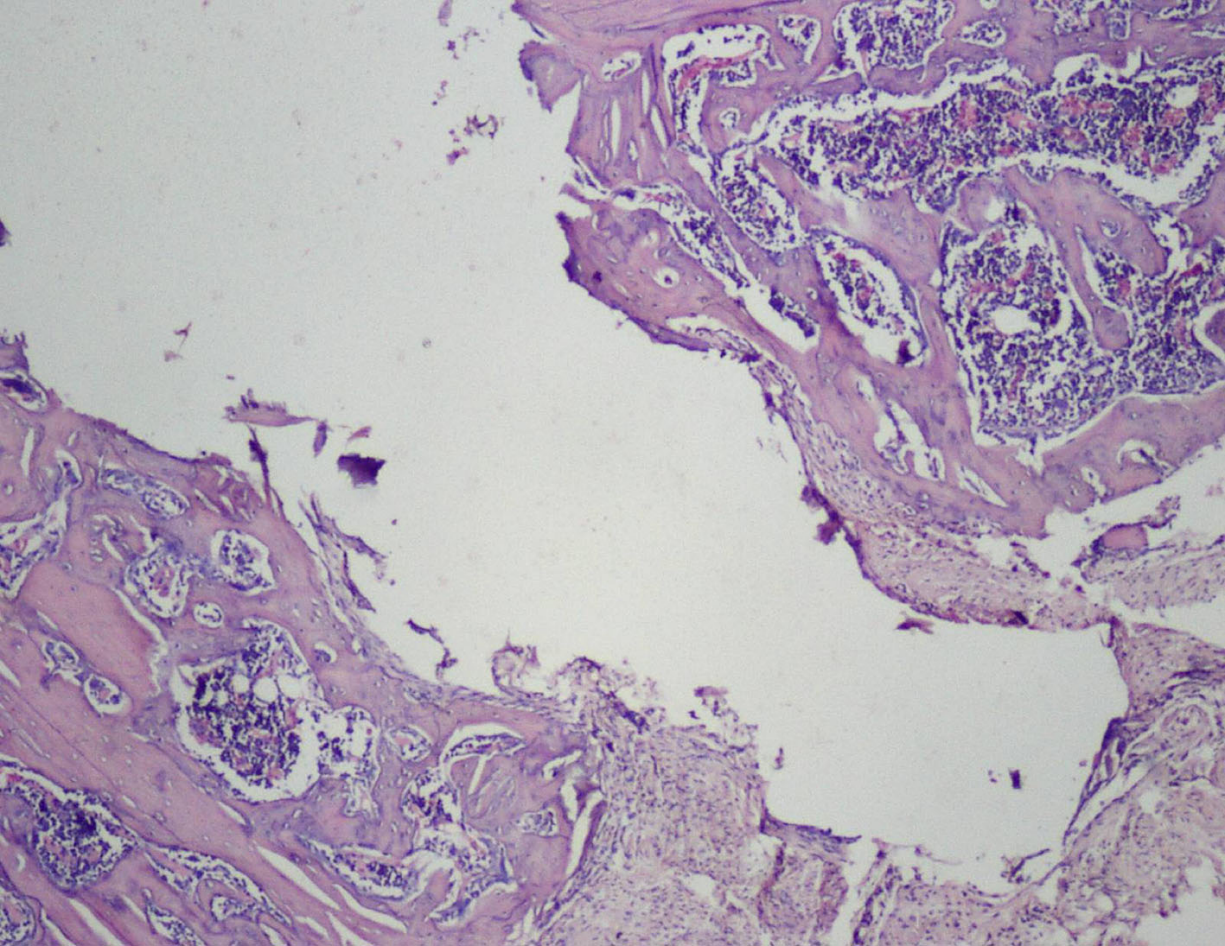
Day 28 of group 4 implant surface with bone formation (photomicrograph of H&E stained slides, original magnification ×10).

Thus, the effect of sodium alendronate alone (Group 2), the combined effect of LLLT and sodium alendronate (Group 3), and the effect of LLLT alone (Group 4) was evaluated and compared with that of controls (Group 1) on 28 ^th^ day, as can be seen in Figure 6, where the implant surface (with bone formation) in Group 3 was treated with sodium alendronate and LLLT, while Figure 7 depicts the implant surface (with bone formation) in Group 4 treated with LLLT alone.

**Figure 8.**
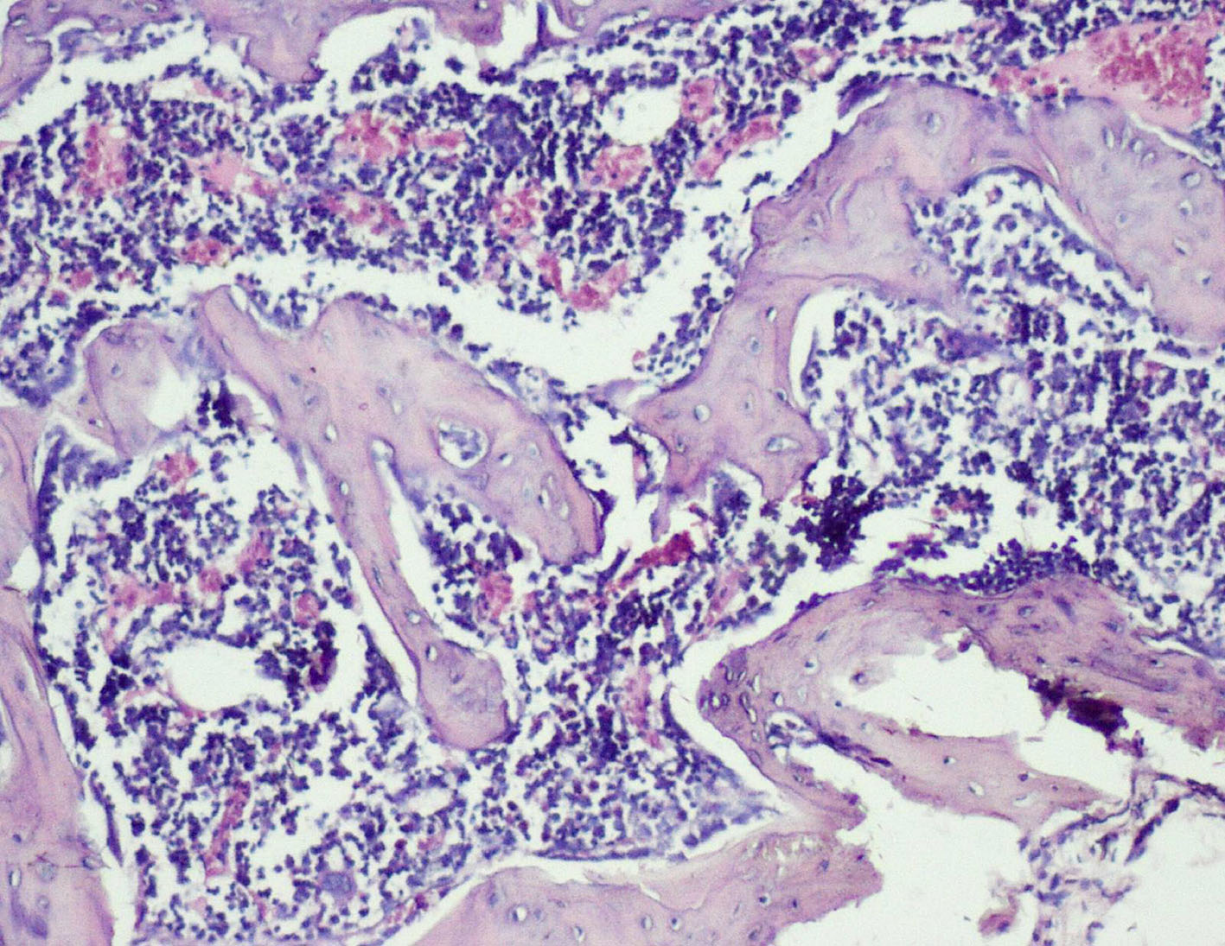
Day 28 of group 3 presence of bony union with reorganized spongiosa formation (photomicrograph of H&E stained slides, original magnification ×10).

**Figure 9.**
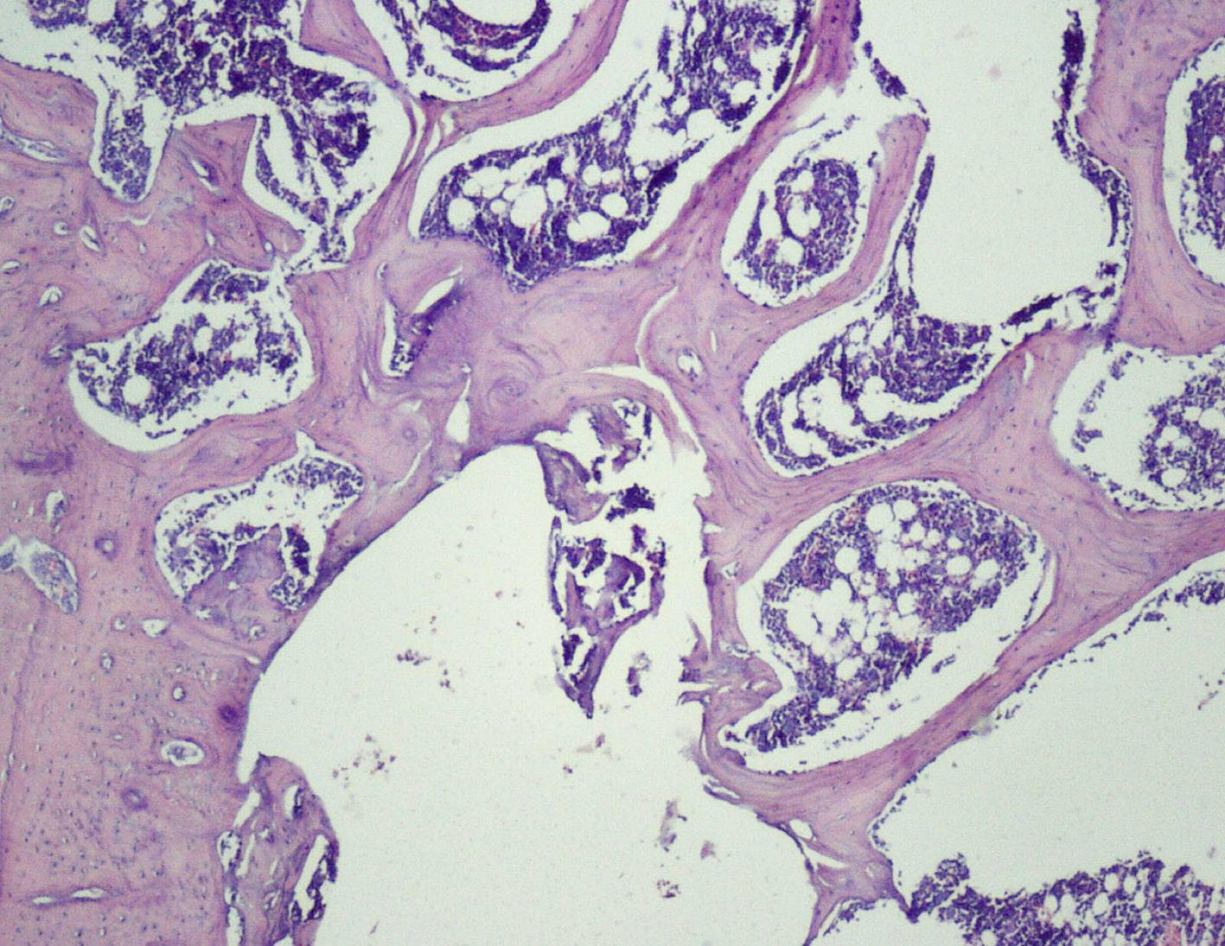
Day 28 of group 3 reorganized spongiosa and bone marrow formation (photomicrograph of H&E stained slides, original magnification ×10).

**Figure 10.**
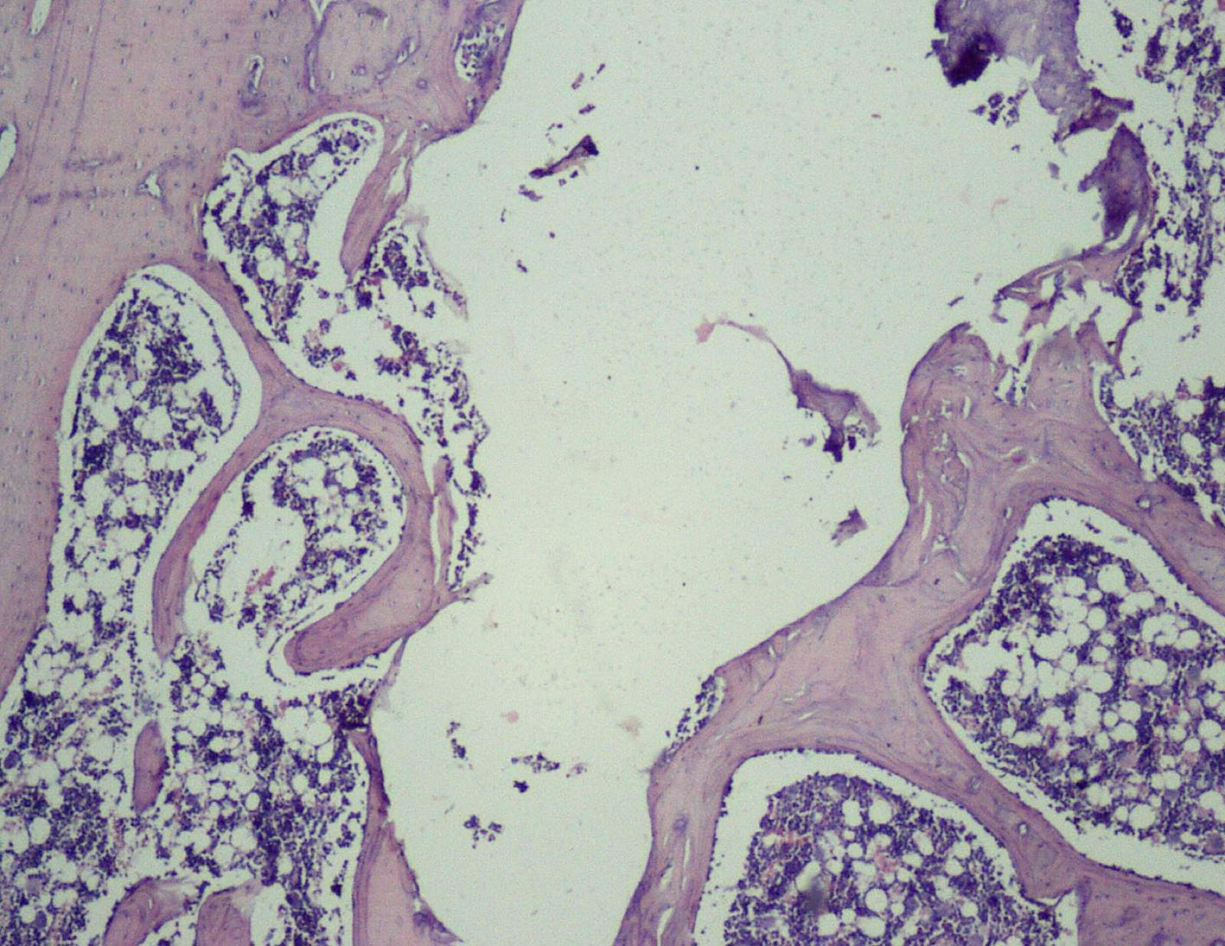
Day 28 of group 4 reorganized spongiosa and bone marrow formation (photomicrograph of H&E stained slides, original magnification ×10).

**Figure 11.**
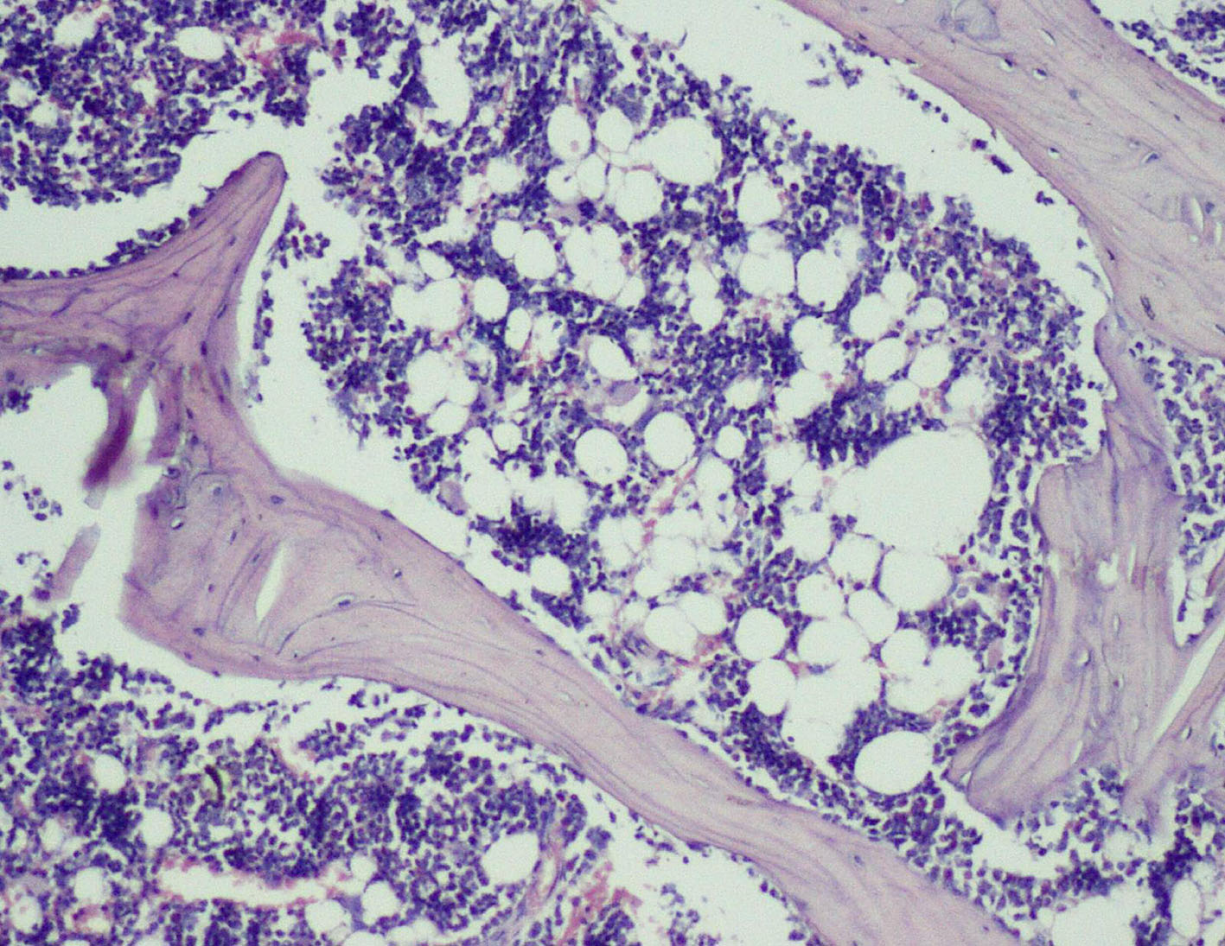
Day 28 of group 3 areas of bone with adult type of fatty marrow (photomicrograph of H&E stained slides, original magnification ×10).

**Figure 12.**
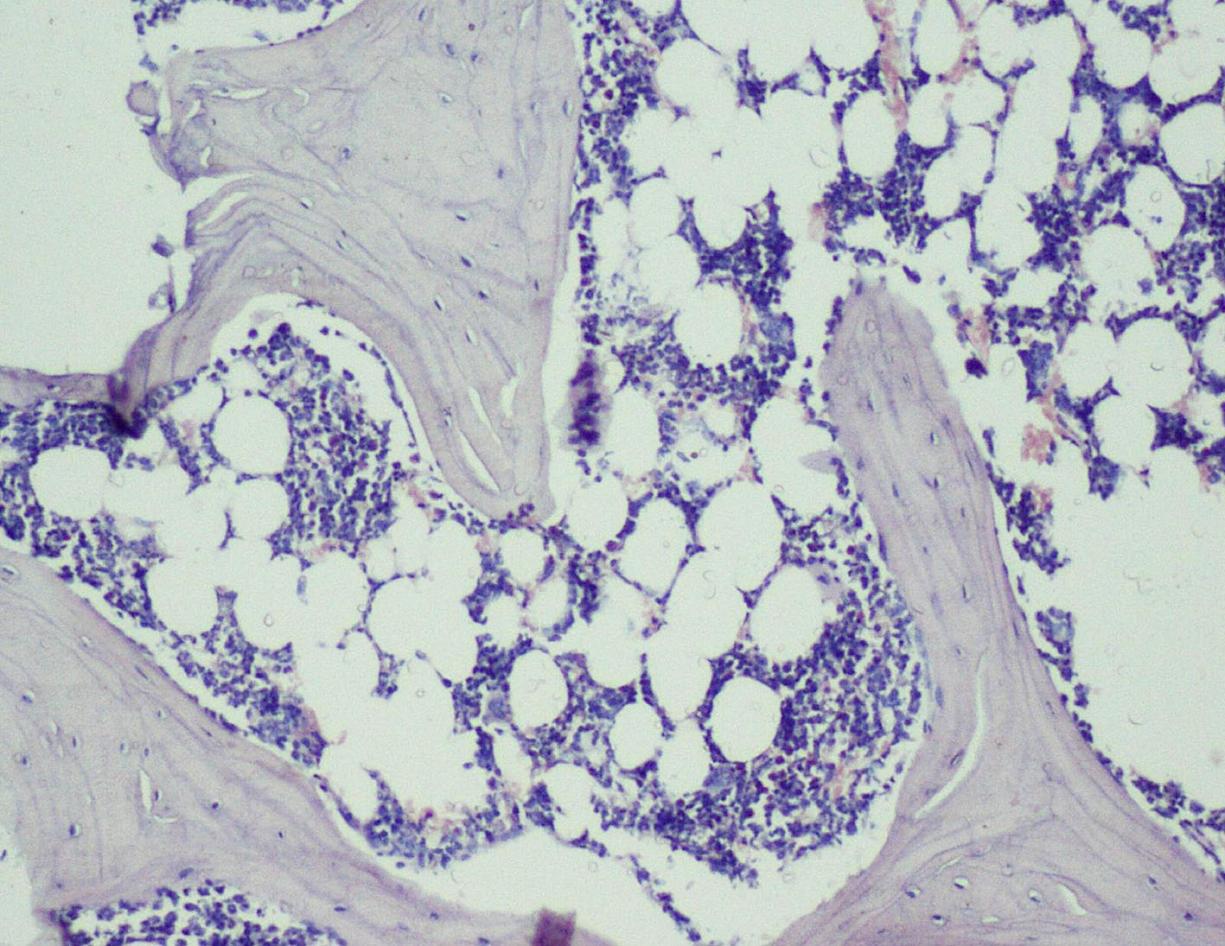
Day 28 group 4 areas of bone with adult type of fatty marrow (photomicrograph of H&E stained slides, original magnification ×10).

**Figure 13.**
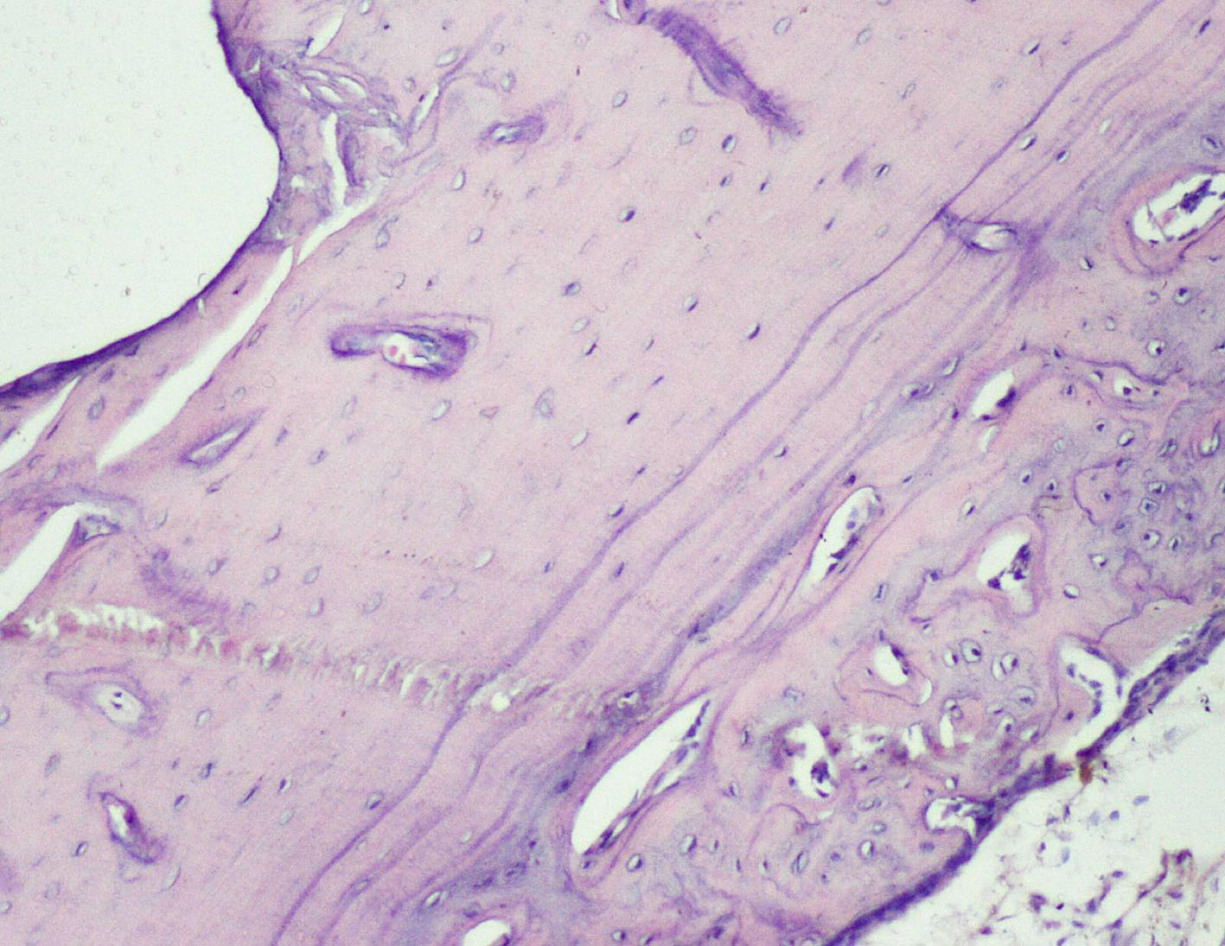
Day 28 of group 3 mature bone formation and reorganization of cortex (photomicrograph of H&E stained slides, original magnification ×10).

**Figure 14.**
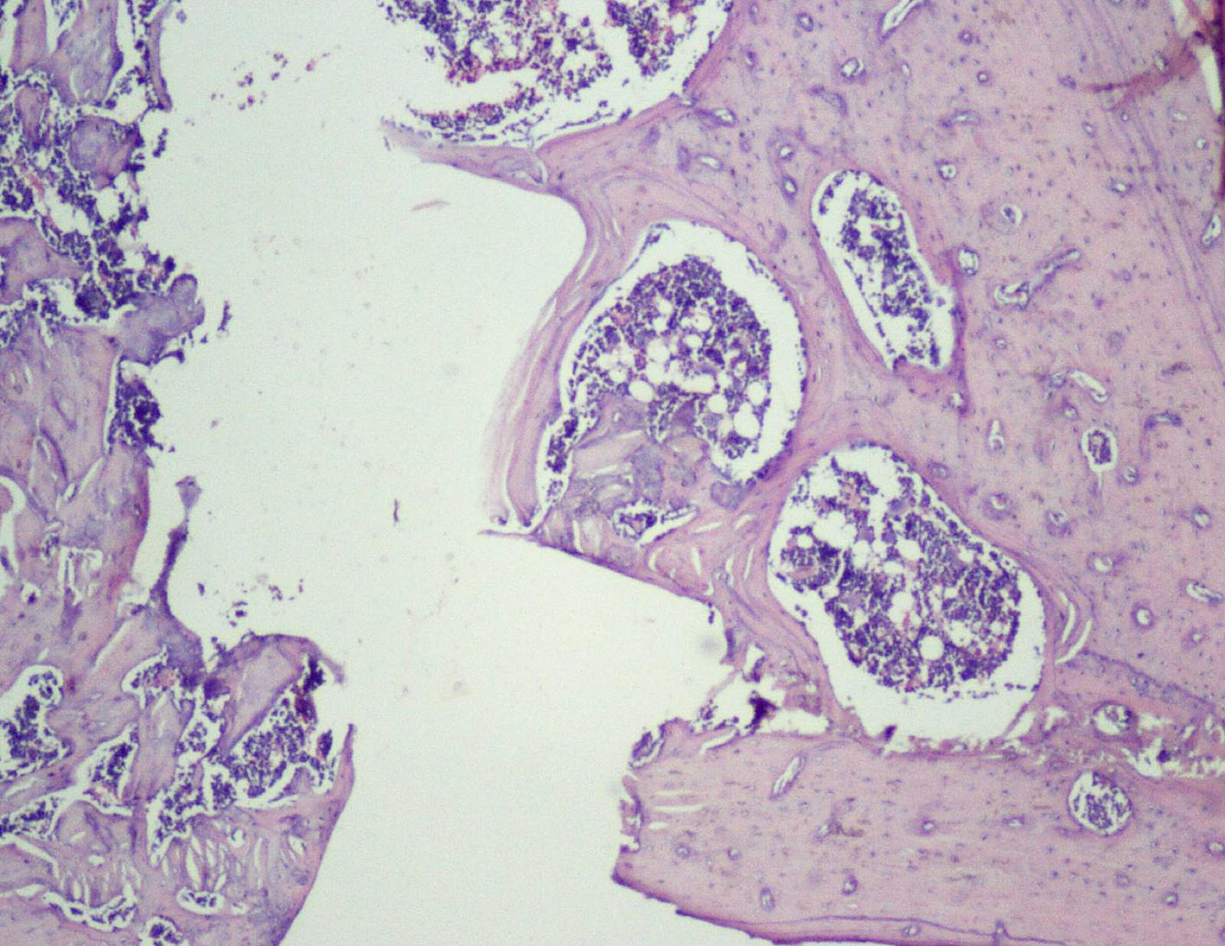
Day 28 of group 4 mature bone formation, marrow spaces and reorganization of cortex (photomicrograph of H&E stained slides, original magnification ×10).

Histological scores of Groups 3 and 4 were statistically significant when compared with Groups 1 and 2 ( Table 2). Presence of bony union with reorganized spongiosa, which is considered one of the important features of favorable healing, was seen in Groups 3 ( Figure 8) and 4, whereas Groups 1 and 2 showed osteochondral union with formation of new bone. Presence of osteo-chondral union after 28 days is considered a sign of unfavorable healing. Reorganization of the cortex was appreciated in all the 4 groups but was significantly higher in Groups 3 ( Figure 9) and 4 ( Figure 10), compared to Group 1 (control); thus healing in Groups 3 and 4 was faster and more favorable in these groups. Adult-type fatty marrow, the presence of which is highly desirable and considered the most important feature of healing, was appreciated in Groups 3 ( Figure 11) and 4 ( Figure 12), whereas it was not appreciated in the control as well as in the Sodium alendronate groups. Thus, a faster healing was present in Groups 3 and 4, compared to Groups 2 and 1. Rats of Groups 3 and 4 were irradiated with LLLT at 7-day intervals and sacrificed on the 28 ^th^ day so that the effect of sodium alendronate combined with LLLT could be evaluated for mature bone formation. It can be seen in both these groups ( Figures 13, 14 respectively) that there is formation of mature bone as well as reorganization of cortex. Thus, LLLT alone or when combined with Sodium alendronate increases formation of new bone.

The sum of histological scores was statistically significant for Groups 3 and 4, compared to Groups 1 and 2; thus we can interpret that Groups 3 and 4 showed better healing than in Groups 1 and 2 after 28 days of implant placement. The Sodium Alendronate group (Group 2) in our study did not yield any statistically significant results but had higher histological scores when compared to Group 1 (control) except for bone marrow formation. Hence, Group 2 showed better healing, compared to the control group. When the histological scores of Groups 3 and 4 were compared, no statistically significant differences were observed between them, but Group 3 had more histological scores; therefore, Group 3 had better healing compared to Group 4. Also, no statistically significant differences could be attributed, when scores between the right and the left side were compared.

## Conclusions

Peri-implant healing of Orthodontic Mini screws placed in Wistar rat femurs could be enhanced by the use of CO _2_ laser therapy, when harnessed at a wavelength of 830 nm, the dose received was about 2.1 J/cm ^2^ (39.2 mW × 10 ^−3^ W × 55 sec) and when combined with 1mg Sodium Alendronate mixed with 1mg Carbapol gel. At the histological level, there is increased formation of reorganized spongiosa and cortical bone as well as enhanced production of adult-type fatty bone marrow.

## Ethical approval

Before commencing the study, clearance was obtained from the Institutional Animal Ethics Committee, Kasturba Medical College Manipal, Karnataka – IAEC/KMC/11/2016.

## Animal ethics

All animal experiments were conducted in compliance with the Institutional Animal Ethics Committee, and the protocol was approved by the same. Efforts were made to minimize animal suffering throughout the study, including the use of appropriate anesthesia. At the conclusion of the experiments, animals were humanely euthanized per ethical guidelines to ensure minimal distress.

## Consent for publication

Not applicable.

## Authors’ contributions

DM and DS realized the research. DM, ASU, ASN, DS was the major contributor in writing the manuscript. DS, LNB, GS helped with the research. ASU, ASN, DS corrected the manuscript. All the authors read and approved the final manuscript.

## Data Availability

Figshare: A Randomized Controlled Trial Evaluating the Effect of Local Application of Sodium Alendronate Gel and Low-Level Laser Therapy (LLLT) On Peri-Implant Tissue Healing in Wistar Rats. Doi: https://doi.org/ 10.6084/m9.figshare.28093763. ^47^ This project contains following underlying data:
• Excel.xlsx Data are available under the terms of the Creative Commons Attribution 4.0 International license (CC-BY 4.0). *Reporting guidelines* ARRIVE guidelines: Doi: https://doi.org/10.6084/m9.figshare.28093763. ^47^ Data are available under the terms of the Creative Commons Attribution 4.0 International license (CC-BY 4.0).
