# Supplementary material for: A Randomized Controlled Trial Evaluating the Effect of Local Application of Sodium Alendronate Gel and Low-Level Laser Therapy (LLLT) On Peri-Implant Tissue Healing in Wistar Rats
Source: F1000Res. 2025 Jan 30;14:147. [Version 1] doi: 10.12688/f1000research.160479.1 (PMC11868745; doi:10.12688/f1000research.160479.1)
Supplement: Peer review history

# Reviewer report: Reviewer response for version 1

**DOI:** 10.5256/f1000research.176384.r364233

**Published:** 2025-02-27

**Authors:** Veerasathpurush Allareddy

**Affiliations:** 1 Department of Orthodontics, University of Illinois Chicago, Chicago, Illinois, USA

Mishra and colleagues evaluate the combined effect of local application of Sodium Alendronate incorporated in Carbapol gel and Low Level Laser Therapy on peri-implant tissue healing in Wistar rats. 24 Wistar rats aged 6 to 7 months were used in this study.

 The authors showed that Peri-implant healing of Orthodontic Mini Implants can be enhanced by use of CO2 laser therapy combined with Sodium Alendronate incorporated in Carbapol gel. This study has much clinical significance and translational potential.

 The authors did an excellent job setting up the context of study and quoting pertinent literature. The methods section clearly explained the experiments conducted and the statistical analyses were appropriate. Overall, a very interesting and informative study.

Is the work clearly and accurately presented and does it cite the current literature?

Yes

If applicable, is the statistical analysis and its interpretation appropriate?

Yes

Are all the source data underlying the results available to ensure full reproducibility?

Yes

Is the study design appropriate and is the work technically sound?

Yes

Are the conclusions drawn adequately supported by the results?

Yes

Are sufficient details of methods and analysis provided to allow replication by others?

Yes

Reviewer Expertise:

Cleft and Craniofacial Orthodontics, Practice-Based Research, and Big Data Analytics.

I confirm that I have read this submission and believe that I have an appropriate level of expertise to confirm that it is of an acceptable scientific standard.

# Reviewer report: Reviewer response for version 1

**DOI:** 10.5256/f1000research.176384.r364236

**Published:** 2025-02-12

**Authors:** Goutham B

**Affiliations:** 1 Department of Orthodontics and Dentofacial Orthopaedics, Coorg Institute of Dental Sciences, Virajpet, Karnataka, India

The study precisely evaluated the impact of Sodium Alendronate (SA) in Carbapol gel and Low-Level Laser Therapy (LLLT) on peri-implant tissue healing in Wistar rats, showing that the combined approach enhanced bone formation compared to individual treatments or controls.

 This study has a well-structured methodology and clear parameters providing potential clinical relevance for orthodontics.

 The study highlights the potential of combining SA and LLLT to optimize peri-implant healing, warranting further investigation for clinical application.

Is the work clearly and accurately presented and does it cite the current literature?

Yes

If applicable, is the statistical analysis and its interpretation appropriate?

I cannot comment. A qualified statistician is required.

Are all the source data underlying the results available to ensure full reproducibility?

Yes

Is the study design appropriate and is the work technically sound?

Yes

Are the conclusions drawn adequately supported by the results?

Yes

Are sufficient details of methods and analysis provided to allow replication by others?

Yes

Reviewer Expertise:

Accelerated orthodontics, Implant dentistry, smile designing

I confirm that I have read this submission and believe that I have an appropriate level of expertise to confirm that it is of an acceptable scientific standard.
